# Screening for infectious diseases and vaccination status in Ukrainian paediatric refugees: a retrospective cohort study

**DOI:** 10.1007/s00431-026-07243-4

**Published:** 2026-07-23

**Authors:** Nina Vaezipour, Sarah Depallens, Noémie Wagner, Irène Frank, Fabienne N. Jaeger, Denise Felber Dietrich, Malte Kohns Vasconcelos, Corinne Wyder, Klara M. Posfay-Barbe, Ulrich Heininger, Nicole Ritz

**Affiliations:** 1 Paediatric Infectious Diseases and Vaccinology, University Children’s Hospital Basel, Basel, Switzerland; 2 Mycobacterial and Migrant Health Research Group, Department of Clinical Research, University of Basel, Basel, Switzerland; 3 Department of Paediatrics and Paediatric Infectious Diseases, Children’s Hospital, Lucerne Cantonal Hospital, Lucerne, Switzerland; 4 Health and Prevention Services, Municipality of Lausanne, Lausanne, Switzerland; 5 Paediatric Infectious Diseases Unit, Department of Woman, Child and Adolescent, Children’s Hospital of Geneva & Faculty of Medicine, Geneva, Switzerland; 6 Clinical Trial Unit, Cantonal Hospital of Lucerne, Lucerne, Switzerland; 7 SwissTPH, Allschwil, Switzerland; 8 University of Basel, Basel, Switzerland; 9 School Health Services, Health Services of the City of Bern, Bern, Switzerland; 10 Department of Epidemiology, Institute for Medical Biometry and Epidemiology, University Medical Centre Hamburg Eppendorf, Hamburg, Germany; 11 Praxis Kinderärzte KurWerk, Burgdorf, Switzerland; 12 Faculty of Medicine, University of Basel, Basel, Switzerland; 13 Migrant Health Reference Group, Paediatrie Schweiz, Fribourg, Switzerland; 14 Erlenhof Migrant Outpatient Clinic, Reinach, Switzerland

**Keywords:** Refugees, Children, Guideline adherence, Infectious disease screening, Ukraine, Real-world data

## Abstract

Paediatric refugees face heightened health risks. At the same time, lack of real-world data hampers the development of evidence-based guidelines for their health care providers. The aim of the study was to assess health-care data and guideline adherence regarding paediatric Ukrainian refugees. Demographic and health-related data were collected from paediatric Ukrainian refugees who arrived in Switzerland between 24 February and 31 October 2022. Data was obtained from five participating centres. Information on infectious disease screening tests and vaccination status was extracted from a REDCap database. Adherence to national guidelines for the care of Ukrainian paediatric refugees was assessed. Of 517 children enrolled, test positivity rates were 2.7% (2/74) for HIV, 1.6% (2/125) for hepatitis B, 0.7% (2/307) for hepatitis C, and 1.0% (3/308) for tuberculosis screening. A high rate of refusal of HIV screening was observed, substantially reducing the intention-to-test rate (0.38–1.25%). Age-appropriate vaccination status was documented in 71–82% of children for measles–mumps–rubella, diphtheria–tetanus–pertussis, poliomyelitis, and *Haemophilus influenzae* type b. Main reason for incomplete vaccination status was vaccine hesitancy. Following arrival in Switzerland, 98% (509/517) of participants completed catch-up vaccination programmes.

*Conclusion*: Our findings highlight challenges in the uptake of recommended infectious disease screening among paediatric refugees, underscoring the need for targeted and culturally sensitive screening strategies. The observed positivity rates provide important data to refine current screening recommendations. Moreover, the marked improvement in vaccination coverage after counselling highlights the effectiveness of addressing vaccine hesitancy in refugee populations.

**Supplementary Information:**

The online version contains supplementary material available at 10.1007/s00431-026-07243-4.

**Table:** 

**What is Known:** • *A substantial proportion of children arriving from Ukraine have incomplete vaccination status, while comprehensive health data on paediatric refugees from Ukraine remain limited.* • *The implementation of and adherence to health care guidelines for paediatric refugees across Europe is highly variable.*
**What is New:** • *Tuberculosis test positivity rates in Ukrainian children who arrived in Switzerland during the early phase of the war were substantially lower than expected, while testing refusal for HIV was high.* • *While prevalence of incomplete vaccination at arrival is high, counselling and catch-up programmes in host countries can be highly effective in rapidly closing vaccination gaps.*

## Introduction

Forced child migration in Europe has increased over the past decade [1], with dynamic shifts in both countries of origin and host nations. This trend is driven by evolving geopolitical situations, climate change, and other factors [2]. Children and adolescents forced to flee are exposed to significant health risks before and during displacement, as well as after arrival in host countries, necessitating specialised health care approaches [3]. Across Europe, the implementation of paediatric refugee health guidelines varies widely, reflecting differences in national policies and health system capacities [4].

Furthermore, migrants across Europe consistently show an elevated risk of incomplete vaccination due to access, legal, and service-level barriers, including the absence of harmonised migrant-specific vaccination guidelines [5]. To address these challenges, national and international guidelines have been developed to support health care providers in the assessment and care of newly arrived children and adolescents in countries of asylum. However, most guidelines face two key limitations. First, recommendations are frequently based on limited scientific evidence, reflecting the overall scarcity of research on the health needs of paediatric migrants [6]. Second, implementation of recommendations in routine health care settings is often inconsistent or inadequate [7]. In a recent study, routine vaccination coverage among Norwegian-born children of Ukrainian refugees was substantially higher than the coverage observed among Ukrainian children who migrated to Norway, underscoring the need to strengthen vaccine uptake and surveillance among newly arrived Ukrainian paediatric refugees [8].

In 2022, members of the Migrant Health Reference Group of Paediatrics Switzerland and the Paediatric Infectious Disease Group in Switzerland (PIGS) developed through a modified Delphi process a guideline for health care providers caring for paediatric refugees from Ukraine, recommending a structured approach including general health assessment, review and catch-up of vaccinations, and targeted screening for tuberculosis, HIV, and hepatitis B and hepatitis C [9]. While this guideline represents an important step towards addressing health needs of paediatric migrants, its development was done with limited available evidence. Consequently, there is a critical need to evaluate both the evidence underpinning such guidelines and their real-world implementations.

The aim of this study was to evaluate adherence to existing guidelines by analysing their implementation in a cohort of paediatric refugees in Switzerland originating from Ukraine. In addition, we evaluated health-related data to determine the relevance of the current recommendations.

## Methods

### Study design and population

A retrospective national, multicentre health-related data collection was performed from February 24th, 2022, to October 31st, 2022, with collection of epidemiological and demographic data from paediatric Ukrainian refugees arriving in Switzerland during this time.

### Database and data collection

Data were collected using REDCap (Research Electronic Data Capture), a web-based platform used to build and manage online databases. Each participating centre entered patient data during or after the clinical visits. After completion of the collection period, the dataset was exported from REDCap for descriptive statistical analysis.

The participating centres included the University Children’s Hospital Basel (UKBB), Basel; the University Children’s Hospital Geneva (Hôpitaux Universitaires de Genève, HUG), Geneva; the Children’s Hospital of Central Switzerland (KidZ), Lucerne; Health Services Bern (Gesundheitsdienste der Stadt Bern), Bern; and a private practice (Praxis Kinderärzte KurWerk), in Burgdorf.

Inclusion criteria were children and adolescents less than 18 years of age who had arrived in Switzerland from Ukraine after 24th of February 2022 and were screened at one of the participating centres before December 31st, 2022. Screening was voluntary except for *the Gesundheitsdienste der Stadt Bern*. All participating children, adolescents and/or their caregivers, signed a written informed consent in Ukrainian language unless the testing and reporting was mandatory.

### Study variables

We collected data on dates of arrival in Switzerland and the first medical visit, alongside anthropometric measurements including weight, height, and body-mass index (BMI), as well as age, sex, and known chronic diseases, defined as pre-existing conditions such as cardiopathy or autism spectrum disorder. Infectious disease screening included serological testing for human immunodeficiency virus (HIV), hepatitis C (HCV), and hepatitis B (HBV)—assessing HBs-antigen, anti-HBs, anti-HBc, and anti-HBe antibodies—as well as tuberculosis (TB) screening using interferon-gamma release assays (IGRA). All tests were performed in routine laboratories at the study centres and interpreted according to recommended cutoff values. Vaccination status was documented through caregiver report, vaccination cards, or other health records when available, complemented by antibody testing for tetanus and measles. Information on referrals to specialists and general practitioners was also collected. Guideline adherence was measured by comparing each child’s documented screening tests and vaccinations with the national recommendations. Missing data are reported where applicable, with corresponding adjustments to denominators as needed.

### Statistical analyses

All statistical analyses were done using the open-source software R (version 4.5.2). Demographic and clinical characteristics were summarised, and descriptive statistics were used to analyse the results for each recommended screening test and vaccination.

### Role of the funding source

The funding organisation was neither involved in the study’s design, data collection, data analysis and interpretation, nor in the preparation, review, or approval of the manuscript.

## Results

### Demographic and baseline characteristics

A total of 517 children and adolescents were included over a 6-month period, 51% of whom were male. The age ranged between 0 and 17 years, with a median of 9 (IQR 5–12) years. The median interval between arrival in Switzerland and first visit at a Swiss health care centre was 64 (IQR 41–92) days. Further baseline characteristics of included children and adolescents are shown in Table 1*.*

**Table 1:** Baseline characteristics of paediatric migrants from Ukraine presenting to primary care services (*n* = 517)

Characteristic	*n* (%) or median (IQR)
**Sex**	
Male	261 (50.6%)
Female	255 (49.4%)
Missing	1 (0.2%)
**Age (years)**	
Range	0–17
Median (IQR)	9 (5–12)
0–1 years	39 (7.5%)
2–4 years	69 (13.3%)
5–12 years	280 (54.2%)
13–17 years	129 (24.9%)
**Bodyweight (kg)**	
Median (IQR)	27.0 (18.7–45.0)
Missing	90 (17.4%)
**Height (cm)**	
Median (IQR)	132.0 (114.0–157.7)
Missing	110 (21.3%)
**Body-mass index (kg/m**^**2**^**)**	
Median (IQR)	16.4 (15.0–18.4)
Missing	110 (21.3%)
**Chronic diseases (n)**	122 (24%)
**Days between arrival and first visit (days)**	
Median (IQR)	64 (41–92)
Missing	17 (3.3%)

Data are median (IQR) unless otherwise stated. Body-mass index (BMI) = weight in kg divided by height in m^2^

**Fig. 1:**
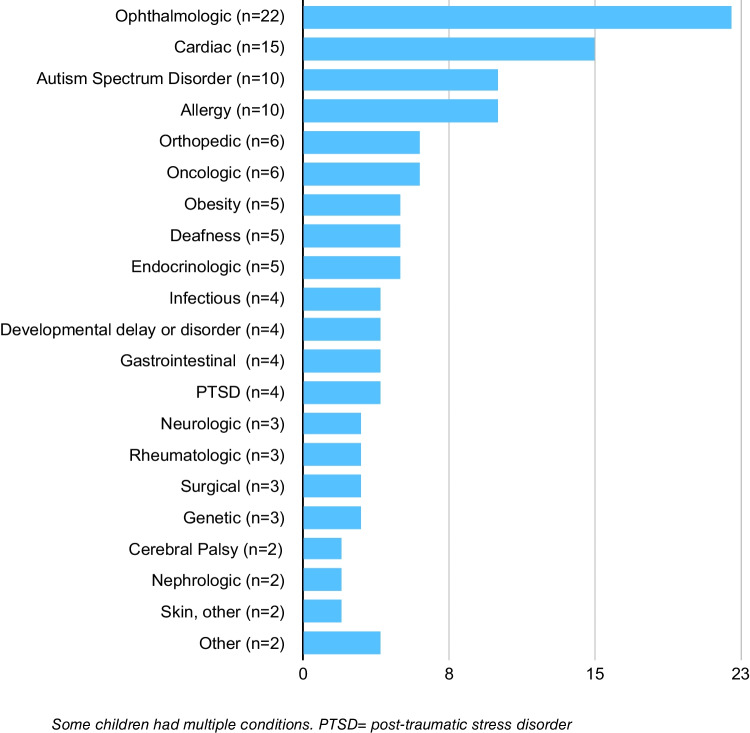
Absolute numbers of paediatric migrants with underlying medical condition by speciality

A total of 24% (122/515) known chronic condition have been reported. The four leading conditions reported were ophthalmological disorders (mainly myopia), cardiac disease (mainly congenital heart defects), autism spectrum disorder, and allergy (not further specified). Post-traumatic stress disorder (PTSD) was stated in 3% (4/122) children. The full list of reported disorders is shown in Fig. 1.

### Referral pathways to health care access and specialists

**Fig. 2:**
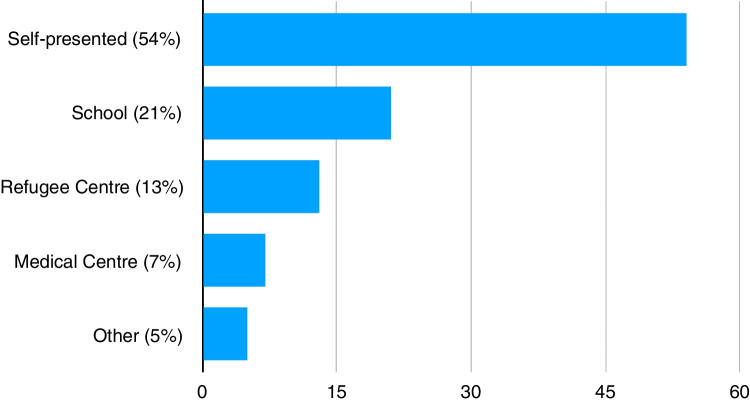
Referral pathways to service among Ukraine paediatric refugees (*n* = 517)

Fifty-four percent of the children and adolescents were self-presenting, and of those, 50% had an acute medical problem, while the other 50% presented for a general screening. Twenty-one percent of children and adolescents were referred from school and 13% from refugee centres (Fig. 2).

After primary assessment, a total of 23% (122/515) were referred to another specialist with the top three referrals being to an ophthalmologist (8%), dentist (6%), and cardiologist (6%). For the full list of referral specialties see Supplementary Table 1.

### Infectious disease screening

Information on performed infectious diseases testing was not available for 26–69% of included children and adolescents, depending on the specific test. Of those with available information, 46% (74/160) had HIV testing done. Testing rates for TB infection were notably higher, 80% (308/384). Similarly, serology testing for HBV and HCV was performed in 62% (125/202) and 81% (307/381) of children and adolescents, respectively. The primary reason for not testing was parental refusal according to reporting clinicians, although this information was not systematically recorded in the database.

Positive test rates for infections with HIV, HBV, HCV, and TB infection were 2.7%, 1.6%, 0.7%, and 1%, respectively (for further details see Table 2).

**Table 2:** Screening tests and positivity rates in paediatric migrants from Ukraine (*n* = 517)

Screening test	Positive *n*/*N* (%)	Tested *n* (%)	Not tested *n* (%)
HIV (p24 antigen and anti-HIV-1/2 antibodies)	2/74 (2.7%)	74 (14.3%)	443 (85.7%)
IGRA (tuberculosis)	3/308 (1.0%)	308 (59.6%)	209 (40.4%)
HBsAg	2/125 (1.6%)	125 (24.2%)	392 (75.8%)
Anti-HBs antibodies	68/105 (65.0%)	105 (20.3%)	412 (79.7%)
Anti-HBc antibodies	1/116 (0.9%)	116 (22.4%)	401 (77.6%)
Anti-HBe antibodies	0/101 (0.0%)	101 (19.5%)	416 (80.5%)
Hepatitis C antibodies	2/307 (0.7%)	307 (59.4%)	210 (40.6%)

Percentages for positive results are calculated among those tested. Percentages for tested and not tested are calculated from the total study population (*n* = 517)

*HIV*, human immunodeficiency virus; *IGRA*, interferon-γ release assay; *HBsAg*, hepatitis B surface antigen; *Anti-HBs*, hepatitis B surface antibody; *Anti-HBc*, hepatitis B core antibody; *Anti-HBe*, hepatitis B e antibody

### Vaccination history and catch-up

The national vaccination programme in Ukraine includes the following vaccines: Bacille Calmette-Guérin (BCG), diphtheria, tetanus, pertussis (DTP) poliomyelitis, *Haemophilus influenzae* type b (Hib), HBV, and measles, mumps, rubella (MMR). Vaccination against varicella, pneumococcal, and meningococcal disease is not included in the routine recommendations (for details see Supplementary Table 2).

Information on vaccination status was available in 92% (474/517) children and adolescents included in the study. The information originated from vaccination cards in 38% (181/474) or from parental recall in 62% (293/474). Vaccinations which are part of the routine vaccination recommendations in Ukraine were best documented or recalled. BCG with 84% and MMR with 82% were the vaccinations with the highest complete coverage. DTP coverage was complete in 71%. The main reason reported for incomplete vaccination status in Ukraine was vaccine refusal in 53%, due to concerns about vaccines in general or about vaccine quality. After consultation at a Swiss health care centre, 98% (509/517) of children and adolescents were up to date according to the Swiss vaccination schedule. Details on vaccinations received in Ukraine and catch-up vaccinations after arrival in Switzerland can be found in Table 3. Additional vaccinations received in Ukraine are shown in Supplementary Table 3.

**Table 3:** Documented or reported vaccinations received in Ukraine and catch-up vaccinations administered in Switzerland (n = 517)

Vaccine	Any vaccination in Ukraine *n*/*N* (%)	Complete vaccination status in Ukraine† *n*/*N* (%)	Any vaccination in Switzerland‡ *n*/*N* (%)
MMR	376/464 (81.0%)	309/376 (82.2%)	98/452 (21.7%)
DTP	391/467 (83.7%)	278/391 (71.1%)	135/457 (29.5%)
HBV	363/467 (77.7%)	288/363 (79.3%)	83/441 (18.8%)
Poliomyelitis	389/465 (83.7%)	287/389 (73.8%)	122/447 (27.3%)
*Haemophilus influenzae* type b	357/461 (77.4%)	266/357 (74.5%)	57/432 (13.2%)
BCG	383/457 (83.8%)	NA	NA
PCV13*	10/78 (12.8%)	8/10 (80.0%)	54/121 (44.6%)
MenACWY*	13/80 (16.3%)	2/13 (15.4%)	60/136 (44.1%)
Varicella*	15/78 (19.2%)	11/15 (73.3%)	25/100 (25.0%)

†Complete vaccination defined according to the Ukrainian national vaccination schedule

‡Catch-up vaccination administered after arrival in Switzerland

*Vaccines not routinely included in the Ukrainian national vaccination programme for all age groups

*MMR*, measles, mumps, and rubella vaccine; *DTP*, diphtheria, tetanus, and pertussis vaccine; *HBV*, hepatitis B virus vaccine; *BCG*, Bacillus Calmette–Guérin vaccine; *PCV13*, 13-valent pneumococcal conjugate vaccine; *MenACWY*, quadrivalent meningococcal conjugate vaccine

Serological testing prior to vaccinations was done in 46% of children for anti-tetanus antibodies and in 40% for anti-measles antibodies and showed high antibody titres for both measles and tetanus (details shown in Supplementary Table 4). Of note, most of the children and adolescents with positive anti-measles antibodies reported to be vaccinated against measles. Only two patients with available measles serologies were previously unvaccinated and showed titres of 469 IU/L and 514 IU/L. Four patients showed positive anti-tetanus titres above or equal to 100 IU/l despite their vaccination documentation, suggesting missing doses. A total of 63% with complete HBV vaccination status had anti-HBs titres above 100 mIU/ml (see Supplementary Table 5).

## Discussion

This study is, to our knowledge, the first to assess the implementation and outcomes of recommended screening approaches among Ukrainian children and adolescents arriving in Switzerland during the early phase of the 2022 war. During this time, Switzerland issued a protection status permit to approximately 60,000 Ukrainian refugees, of whom about one-third were children [10]. The participating centres—three major paediatric hospitals and two refugee reception structures in the German- and French-speaking regions—therefore captured only a subset of the national paediatric refugee population, although they represent a substantial proportion of the country’s paediatric acute care capacity. Current national recommendations include universal screening for HCV and TB in all children and a risk-based recommendation for HIV screening [9]. Our findings indicate a substantial gap in implementation, with only two thirds of children screened for HCV and TB and 14% screened for HIV. These results are consistent with recently published findings from a national survey amongst primary care paediatricians, which identified substantial barriers to the implementation of national screening recommendations [9]. Moreover, the observed prevalence of TB infection and HCV, determined by IGRAs and serology, positivity rates were lower than expected. These findings suggest that, for this population, current systematic or targeted screening strategies may require reconsideration.

In our cohort, the positivity rate of HCV infection was 0.7%. Population-level data from Ukraine suggest a higher estimated prevalence in the general population with 3% [11]. In Ukrainian children and adolescents, however, prevalence of HCV infection remains unclear, while the estimated global prevalence of HCV infection in children and adolescents is 0.15% [12]. Considering the fivefold higher test positivity observed in our cohort compared to global estimates for children and adolescents and considering the potential for HCV infection to cause chronic liver damage, the recommendation for screening should be maintained despite low positivity rates. This is further supported by the availability of highly effective direct-acting antiviral medicines which can lead to cure in a large proportion of individuals with HCV.

The incidence of tuberculosis in Ukraine has been reported to be 73/100,000 persons and is one of the highest in the WHO European region [11]. Therefore, it was expected that the arrival of a large number of Ukrainians in many European countries would considerably increase the number of detected TB cases. This was confirmed in a recent study where authors reported a positive IGRA screening of 13% (597/4677, 95% CI 11.8–13.7) in Ukrainian refugees older than 15 years of age arriving in Germany [13]. However, a German cross sectional study identified positive IGRAs in 13% (*n* = 184) of the adults and only in 2% (*n* = 7) of the children out of 1793 refugees [14]. In our setting, the test positivity rate was even lower, at approximately 1%. This finding may be explained by the arrival of a selected population of Ukrainian families with a potentially lower exposure to tuberculosis during the early phase of the war. The low positivity rate raises questions about the continued justification of universal tuberculosis screening in the Swiss recommendations. In our setting, cost-effectiveness—integrating the prevalence of positive IGRA results and the risk of disease progression—has been evaluated [15]. At a 1% prevalence of a positive IGRA, a TB progression rate of over 25% is required for a screening recommendation to be cost effective. Such high progression rates are only seen in children < 5 years of age [15], and therefore, the recommendation may need to be adjusted.

The positivity rate of HIV was 2.7% (2/74) in our cohort. Of note, one of these two patients was known to have congenital HIV and was referred to an infectious disease specialist. Ukraine has one of the highest HIV burdens in Europe. By May 2022, a modelling study estimated that more than 86,000 forcibly displaced Ukrainians were living with HIV [16]. In 2019, HIV prevalence among the general population in Ukraine was estimated to be about 1%, which is over five times higher than the prevalence in the EU/EEA (0.17%) [17]. The true prevalence of HIV in children and adolescents in Ukraine is unclear, but national data indicated in 2016 that there were about 3000 children and adolescents in the Ukraine living with HIV [18]. Nonetheless, the large displacement of people from Ukraine to EU/EEA countries in 2022 led to a tenfold increase in HIV diagnoses in adults and children in 2022 compared to the previous year (*n* = 223 versus *n* = 2338) [17].

Nevertheless, the prevalence of HIV infection among Ukrainian children arriving in Switzerland may have been overestimated because of selection bias and the small number of tests performed. In many cases, HIV serology was not done either because it was not considered medically indicated in accordance with national recommendations or because of parental refusal, which might be related to HIV-associated stigma in Ukraine [19]. Yet, given the serious consequences of HIV infection, the current recommendation for selective screening—implemented in the absence of a reliable negative maternal HIV test during pregnancy or in the presence of potential exposure or risk factors such as forced migration—appears justified.

For chronic HBV infection, the positivity rates in our study were also relatively high with 1.6% (2/125) HBsAg positivity. One of these two patients had known perinatal HBV exposure, the other patient has been reported as healthy. Both patients had a complete vaccination status regarding HBV vaccination according to the Ukrainian vaccination schedule.

Overall, the HBsAg prevalence in Ukraine is estimated to be 0.7–1% [11, 20]. Ukraine is considered a country with intermediate HBV prevalence, although the prevalence in children is considered to be low with HBsAg prevalence below 0.5% [20]. Over- or underestimation may not be excluded from our data as the number of tested children was only about one-fifth of the total number of children and adolescents in our study. However, the data suggests that the prevalence of HBV might be higher than expected, especially in HBV-unvaccinated children and adolescents.

Consistent with evidence that tailored, culturally sensitive strategies can overcome vaccination inequities [5], most of the vaccination gaps we identified among Ukrainian migrants were successfully closed after arrival in Switzerland through systematic catch-up efforts and accessible immunisation services.

For HBV, approximately 80% of individuals had completed their vaccination prior to consultation. This proportion was higher than expected based on national vaccination coverage in Ukraine, which is reported to be as low as 28.8% [21]. In a seroprevalence study published in 2017, the weighted percentages in children born between 2010 and 2015 who received three or more doses of hepatitis B vaccine varied between 28 and 57% [20]. Similarly, low HBV immunity has been reported among Ukrainian children and adolescents arriving in Poland, where only 65% demonstrated protective anti-HBs antibody levels [22]. Importantly, in our study, nearly all children and adolescents accepted and received catch-up HBV vaccinations following consultation.

Measle vaccination coverage, similar to that of HBV, was above 80% based on the vaccination information provided. This aligns with national recommendations, which do not advise routine measles serology testing [9]. The interpretation of measles serology requires caution, because antibody titres may be falsely negative in vaccinated individuals, rendering the results mainly informative for unvaccinated children. In our cohort, a subset of children underwent serological testing, and most positive titres were observed in those who had received at least one dose of MMR vaccine in Ukraine.

For tetanus, national recommendations suggest determining the need for catch-up vaccinations 4 weeks after a single dose of a tetanus toxoid-containing combined vaccine [9]. In our study, in over 60% of cases, vaccination status was based on parental recall. Interestingly, four children with positive anti-tetanus titres were reported as unvaccinated against tetanus. Maternal antibodies cannot explain these findings, as the children were aged 2–8 years. It is therefore possible that vaccination history was not recalled by caregivers or was not documented.

Our findings should be interpreted in the context of potential temporal and geographic heterogeneity. Health status and vaccination histories of Ukrainian child refugees arriving in later phases of the conflict or in other host countries may differ due to varying pre-departure health conditions and changing migration routes. This cohort represents Ukrainian children and adolescents who arrived during the first 6 months of the war, when migration flows were dominated by families with specific characteristics; subsequent refugee cohorts may differ in socioeconomic status and access to health care prior to departure. We cannot exclude that the relatively low prevalence of HIV and TB and the high vaccination uptake after counselling partially reflect the characteristics of this early-arriving cohort and the Swiss reception context.

In summary, our current Swiss guideline for Ukrainian children and adolescents was only partially followed in routine practice, underscoring the implementation gap between written recommendations and real-world clinical settings. The cohort data allowed us to identify that vaccination counselling and catch-up were highly feasible and that the high rate of testing refusal will require adaptations through direct and repeated counselling on risk and stigma for HIV and hepatitis B infections.

One limitation is the sample size of 517 children, which might have led to an increased variability in results. Furthermore, in some regions of Switzerland, all Ukrainian refugees have been systematically referred for screening purposes, while in others, referral was only done in case of health problems. In addition, reasons for not performing the recommended infectious disease screening tests were not recorded in a standardised manner. Despite these limitations, our study includes the best currently available real-world data in Switzerland relevant for infectious diseases and vaccination coverage among this vulnerable and understudied population. Guidelines for refugee child health must be adaptable to rapid changes in migration flows or resource constraints. Our findings provide evidence for refining existing guidelines intended for health care professionals. Particularly, they suggest that robust vaccination counselling and easy access to free vaccines can rapidly close vaccination gaps, while guideline implementation needs active support rather than passive dissemination alone.

Future prospective longitudinal studies are needed to clarify risk factors underlying these trends, including socioeconomic inequalities, and to assess both physical and mental health outcomes. Ongoing surveillance is essential to ensure that recommendations remain evidence-based and contextually appropriate. Vaccine hesitancy should be systematically monitored and addressed among migrant populations. Linking cohorts across European countries in a unified approach is needed to allow assessment of geographical variation and will help to differentiate context-specific from generalisable findings. In parallel, the development of an interoperable, Europe-wide electronic vaccination registry should be urgently prioritised.

## Supplementary Information

Below is the link to the electronic supplementary material.

Supplementary file1 (DOCX 33.4 KB)

## Data Availability

The individual-level data supporting the findings of this study are available from the corresponding author upon reasonable request for a period of up to five years following publication.
