# Supplementary material for: Screening for infectious diseases and vaccination status in Ukrainian paediatric refugees: a retrospective cohort study
Source: Eur J Pediatr. 2026 Jul 23;185(8):602. doi: 10.1007/s00431-026-07243-4 (PMC13395900; doi:10.1007/s00431-026-07243-4)
Supplement: Supplementary file 1 — Supplementary file1 (DOCX 33.4 KB) [file 431_2026_7243_MOESM1_ESM.docx]

**Supplementary Tables**

**Supplementary Table 1: Referrals to medical specialists in Switzerland after arrival (n=517)**

| **Specialist** | **n/N (%)** |
| --- | --- |
| Ophthalmologist | 40/508 (7·9%) |
| Cardiologist | 28/508 (5·5%) |
| Dentist | 28/508 (5·5%) |
| Neurologist | 23/508 (4·5%) |
| Psychologist | 18/508 (3·5%) |
| Orthopaedist | 16/508 (3·1%) |
| Physiotherapist | 12/508 (2·4%) |
| Endocrinologist | 10/508 (2·0%) |
| Infectious disease specialist | 8/508 (1·6%) |
| Surgeon | 7/508 (1·4%) |
| Haemato-oncologist | 4/508 (0·8%) |

**Supplementary Table 2: National vaccination programme in Ukraine (adapted from official National Vaccination Schedule)***

| **Vaccine** | **2 days** | **2 months** | **4 months** | **6 months** | **12 months** | **18 months** | **4**  **years** | **6 years** | **12-13 years** | **16 years** |
| --- | --- | --- | --- | --- | --- | --- | --- | --- | --- | --- |
| Hepatitis B | – | 1st  dose | 2nd dose | 3rd dose | – | 4th  dose |  | – | – | – |
| BCG | 1st dose | – | – | – | – | – |  | – | – | – |
| MMR | – | – | – | – | 1st dose | – | 2nd dose |  | – | – |
| DTP | – | 1st dose | 2nd dose | 3rd dose | – | 4th  dose |  | 5th dose (DT) | – | 6th dose (DT) |
| Hib |  | 1st dose | 2nd dose | 3rd dose |  | 4th  dose |  |  |  |  |
| IPV | – | 1st dose | 2nd dose | 3rd dose |  | 4th  dose |  | 5th dose |  |  |
| HPV |  |  |  |  |  |  |  | 1st dose |  |  |

** Adapted from the Ukrainian National Vaccination Schedule (Ministry of Health), updated March 5th, 2026 (https://moz.gov.ua/uk/immunization)*

*DTP=diphtheria, tetanus and pertussis vaccine; Hib=Haemophilus influenzae type b vaccine; BCG=Bacillus Calmette–Guérin vaccine; MMR=measles, mumps, and rubella vaccine, HPV= human papillomavirus vaccine*

**Supplementary Table 3: Documented or reported additional vaccines received in Ukraine (n=517)**

| **Administered vaccine** | **n/N (%)** |
| --- | --- |
| COVID‑19 | 3/515 (0·6%) |
| Hepatitis A | 4/515 (0·8%) |
| Human papillomavirus (HPV) | 15/515 (2·9%) |
| Influenza | 0/515 (0·0%) |
| Meningococcus group B | 0/515 (0·0%) |
| Rabies | 0/515 (0·0%) |
| Rotavirus | 4/515 (0·8%) |
| Tick-borne encephalitis | 0/515 (0·0%) |
| Yellow fever | 0/515 (0·0%) |

*HPV=human papillomavirus vaccine*

*“0·0%” indicates vaccine not administered in this cohort*

**Supplementary Table 4: Screening results for vaccination‑related serum antibodies (n=517)**

| **Antibody** | **Titer median (IQR) (IU/l)** | **Titer in unvaccinated children (IU/l)** | **Reference value (IU/l)** | **Tested n (%)** | **Not tested n (%)** | **No information n (%)** |
| --- | --- | --- | --- | --- | --- | --- |
| Anti-tetanus antibodies | 1289 (219–3392) | 250; 1723; 100; 100¹ | ≥1000 | 84/130 (46·2%) | 99/183 (53·8%) | 334/517 (64·6%) |
| Anti-measles antibodies | 609 (300–994) | 469; 514^1^ | ≥500 | 65/163 (39·9%) | 98/154 (60·1%) | 354/517 (68·5%) |

1. *Each value represents the result for an individual child*

**Supplementary Table 5: Hepatitis B vaccination status and anti-HBs values**

| **HBV vaccination status** | **Anti-HBs <10 mIU/ml** | **Anti-HBs 10–99 mIU/ml** | **Anti-HBs ≥100 mIU/ml** | **Anti-HBc positive** | **Anti-HBe positive** |
| --- | --- | --- | --- | --- | --- |
| Complete (n=16) | 1/16 (6·3%) | 5/16 (31·3%) | 10/16 (62·5%) | 0/16 (0·0%) | 0/16 (0·0%) |
| Incomplete (n=45) | 1/45 (2·2%) | 15/45 (33·3%) | 29/45 (64·4%) | 1/45 (2·2%) | 0/45 (0·0%) |

*Anti-HBs = hepatitis B surface antibody; anti-HBc = hepatitis B core antibody; anti-HBe = hepatitis B e antibody.*

*Percentages calculated from row total (n=16 for complete; n=45 for incomplete)*
